# Cell specialization in cyanobacterial biofilm development revealed by expression of a cell-surface and extracellular matrix protein

**DOI:** 10.1038/s41522-023-00376-6

**Published:** 2023-03-02

**Authors:** Alona Frenkel, Eli Zecharia, Daniel Gómez-Pérez, Eleonora Sendersky, Yevgeni Yegorov, Avi Jacob, Jennifer I. C. Benichou, York-Dieter Stierhof, Rami Parnasa, Susan S. Golden, Eric Kemen, Rakefet Schwarz

**Affiliations:** 1 The Mina and Everard Goodman Faculty of Life Sciences, Bar-Ilan University, 5290002 Ramat-Gan, Israel; 2 Center for Plant Molecular Biology (ZMBP), University of Tübingen, 72074 Tübingen, Germany; 3 Division of Biological Sciences, University of California, San Diego, La Jolla, CA 92093 USA; 4 Center for Circadian Biology, University of California, San Diego, La Jolla, CA 92093 USA

**Keywords:** Biofilms, Microbial ecology

## Abstract

Cyanobacterial biofilms are ubiquitous and play important roles in diverse environments, yet, understanding of the processes underlying the development of these aggregates is just emerging. Here we report cell specialization in formation of *Synechococcus elongatus* PCC 7942 biofilms—a hitherto unknown characteristic of cyanobacterial social behavior. We show that only a quarter of the cell population expresses at high levels the four-gene *ebfG*-operon that is required for biofilm formation. Almost all cells, however, are assembled in the biofilm. Detailed characterization of EbfG4 encoded by this operon revealed cell-surface localization as well as its presence in the biofilm matrix. Moreover, EbfG1-3 were shown to form amyloid structures such as fibrils and are thus likely to contribute to the matrix structure. These data suggest a beneficial ‘division of labor’ during biofilm formation where only some of the cells allocate resources to produce matrix proteins—‘public goods’ that support robust biofilm development by the majority of the cells. In addition, previous studies revealed the operation of a self-suppression mechanism that depends on an extracellular inhibitor, which supresses transcription of the *ebfG*-operon. Here we revealed inhibitor activity at an early growth stage and its gradual accumulation along the exponential growth phase in correlation with cell density. Data, however, do not support a threshold-like phenomenon known for quorum-sensing in heterotrophs. Together, data presented here demonstrate cell specialization and imply density-dependent regulation thereby providing deep insights into cyanobacterial communal behavior.

## Introduction

Cyanobacteria are highly abundant photosynthetic prokaryotes that occupy diverse habitats. These microorganisms are responsible for ~25% of the global CO_2_ converted to organic material and the accompanying O_2_ released in the photosynthetic process^1,2^. Frequently, these photosynthetic prokaryotes are found in microbial assemblages known as biofilms or part of laminated biofilms, dubbed microbial mats^3–5^. Phototrophic biofilms are often associated with industrial problems^6–8^; in contrast, such microbial consortia are beneficial, e.g., for effective biomass accumulation for the biofuel industry and for harvesting of secondary metabolites^9–12^. In-depth understanding of cyanobacterial biofilm development paves the way for inhibition of deleterious biofilms and promotion of beneficial ones.

The mechanisms involved in cyanobacterial aggregation or biofilm formation started emerging only in recent years. For example, similarly to heterotrophic bacteria, cyanobacteria use the second messenger cyclic-di-GMP for regulating aggregated vs planktonic mode of growth^13^. Furthermore, the thermophilic cyanobacterium *Thermosynechococcus vulcanus* employs cyanobacteriochrome photoreceptors to mediate light-color input for controlling cell aggregation via c-di-GMP signaling^14–16^.

Microbial cells within biofilms are encased in a self-produced matrix of hydrated extracellular polymeric substances (EPS) that allows multilayering of cells and structural stability and provides a protected environment. Numerous studies in diverse heterotrophic bacteria identified particular sugar polymers and protein filaments as matrix components^17,18^, however, little is known about the cyanobacterial biofilm matrix. Yet, extracellular polysaccharides were implicated in cyanobacterial biofilm formation, for example, studies of *Synechocystis* support involvement of extracellular polysaccharides in surface adhesion^19^ and cell sedimentation^20^ and cellulose accumulation is responsible for cell aggregation in *T. vulcanus* RKN^21^. The exo-protein HesF of *Anabaena* sp. PCC 7120 is required for aggregation and it was proposed that it interacts with polysaccharides^22^, however, its detailed role in aggregation is still unknown.

**Fig. 1:**
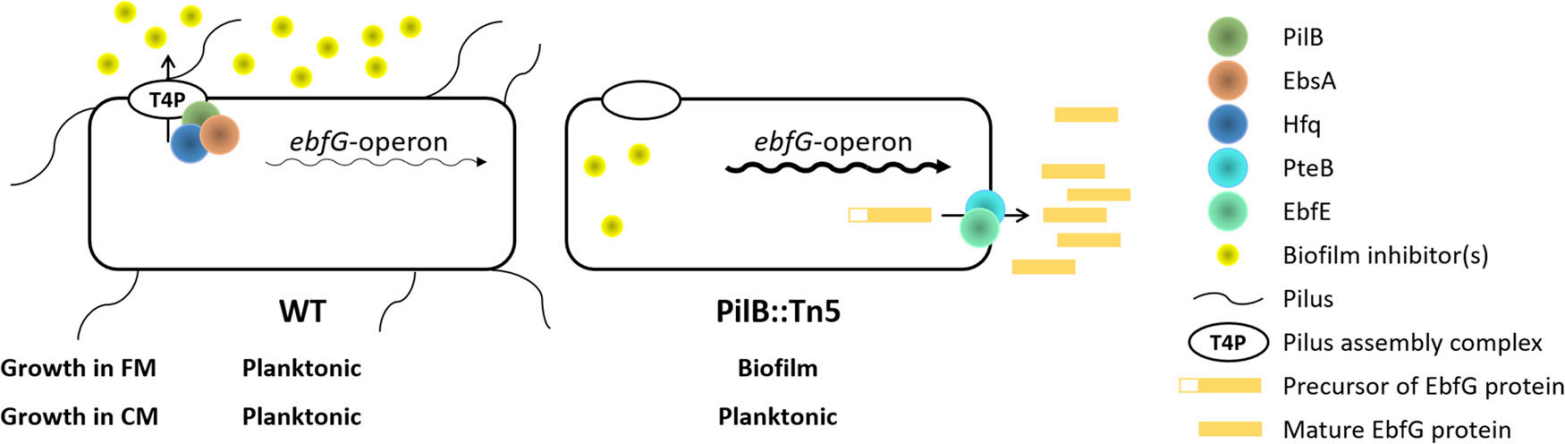
Biofilm regulation in *S. elongatus* by an extracellular inhibitor that dictates transcription of the *ebfG*-operon. The type 4 pilus (T4P) assembly complex is involved in deposition to the extracellular milieu of biofilm inhibitor(s), which dictate transcription of the *ebfG*-operon. Low and high abundance of transcripts of this operon are indicated by thin and thick arrows, respectively. FM fresh medium, CM conditioned medium.

Our previous studies revealed a biofilm self-suppression mechanism in *S. elongatus* that dictates planktonic growth of this strain (Fig. 1). Inactivation of gene Synpcc7942_2071, abrogates the biofilm inhibitory process and results in a biofilm-proficient strain in contrast to the planktonic nature of WT (supplementary video). This gene encodes a homologue of ATPases of type 2 secretion (T2S) systems or type 4 pilus (T4P) assembly complexes, thus, the mutant was initially designated T2EΩ but recently renamed PilB::Tn5^23–25^. The RNA chaperone Hfq and a conserved cyanobacterial protein (EbsA), which are part of the T4P complex, are also essential for the biofilm suppression mechanism^25^. In addition, we identified four small proteins, each with a double glycine secretion motif, that enable biofilm formation (enable biofilm formation with a GG motif EbfG1-4).

The T4P complex is involved in deposition to the extracellular milieu of a biofilm inhibitor, a small MW (<3 kDa), heat stable and protease insensitive molecule, which has not yet been resolved to a specific compound^23,26^. This inhibitor serves for repression of the *ebfG-*operon, which is highly expressed in the PilB::Tn5 strain, where the biofilm inhibitory mechanism is abrogated. Expression is, however, low in WT cultures and in PilB::Tn5 cells inoculated into conditioned medium (CM) from a WT culture^23,26^. Mass-spectrometry (MS) analyses revealed the presence of EbfG1-4 in CM of PilB::Tn5^26,27^. Furthermore, we demonstrated that proteins PteB (peptidase transporter essential for biofilm formation), which belongs to the C39 peptidases family, and EbfE (enable biofilm formation enzyme), a homolog of microcin processing peptidases, are implicated in secretion of EbfG1-4 to the extracellular milieu^26,28^. The role of EbfG1-4 in biofilm formation, however, was unknown. Here, using a reporter construct we demonstrate that high expression of the *ebfG*-operon is limited to a small subpopulation of cells of PilB::Tn5. Further characterization indicates cell-surface and biofilm-matrix localization of EbfG4 and strongly supports amyloid nature of EbfG2. Together, the data indicate cell specialization and imply microbial cooperation for production of extracellular components beneficial for the whole population, known as “public-goods”. In addition, the response of the reporter strain to conditioned media harvested at different stages of logarithmic growth of the wild-type (WT) strain suggests a density dependent mechanism in regulation of *S. elongatus* biofilm development.

## Results

### *ebfG*-operon expression in individual cells

**Fig. 2:**
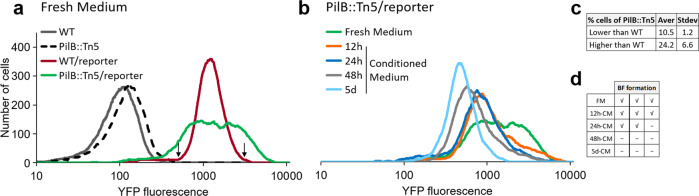
Analysis of expression of the *ebfG*-operon by flow cytometry using reporter strains. **a** Number of cells as a function of fluorescence in cultures grown in fresh medium (FM) for 6 days. Strains analyzed: WT, PilB::Tn5 and their cognate reporter strains that bear a fusion of the regulatory region of the *ebfG*-operon with a yellow fluorescence protein (YFP). Arrows indicate fluorescence cutoff for calculating mutant cells with lower or higher expression of *ebfG*-operon compared to WT. **b** Number of cells as a function of fluorescence in PilB::Tn5/reporter cells grown in FM and conditioned medium (CM). **c** Fraction of PilB::Tn5/reporter cells with lower or higher expression of *ebfG*-operon compared to WT/reporter. Shown are averages and standard deviations from three independent experiments. **d** Biofilm (BF) formation by PilB::Tn5/reporter cells grown in FM or CM harvested at different time points of WT culture.

Previous quantitative RT-PCR analyses indicated that the *ebfG*-operon is highly transcribed in PilB::Tn5 compared to WT^26^. These data reflect the averaged transcription level, thus, to gain insight into variation within the population, here we employ a reporter construct to follow expression of this operon in individual cells. To this end, a DNA fragment bearing the putative *ebfG*-operon promoter along with the 5’ untranslated region was attached to a yellow fluorescence protein (*yfp*) gene, yielding the construct P-*ebfG*::YFP (Supplementary Table 1). This fusion product was inserted in a neutral site 1 in the chromosome in WT and PilB::Tn5 cells yielding WT/reporter and PilB::Tn5/reporter strains, respectively, which were analyzed by flow cytometry (Fig. 2a, b).

Mean and median values of YFP fluorescence level is different between reporter strains and strains lacking the reporter construct, whereas these parameters are similar in WT/reporter and PilB::Tn5/reporter strains grown in fresh medium (Supplementary Fig. 1a). In contrast, data dispersion is larger in PilB::Tn5/reporter compared to WT/reporter (Fig. 2a), a feature that is manifested in the significantly different robust coefficient variation (rCV, Supplementary Fig. 1a). Moreover, data analysis revealed that on average, ~10% of PilB::Tn5/reporter cells are characterized by lower—and ~24% by higher—expression of the *ebfG*-operon (Fig. 2c). This observation suggests cell specialization in *S. elongatus* biofilm development.

Our previous studies demonstrated higher transcription of the *ebfG*-operon in PilB::Tn5 compared to WT when strains are cultured in fresh medium. Inoculation, however, of the mutant into conditioned medium (CM) from WT culture, strongly suppresses transcription^23,26^. These data suggest involvement of intercellular communication in *S. elongatus* biofilm development possibly via a density dependent mechanism. To monitor the presence of the biofilm inhibitor along culture growth, CM was harvested from WT cultures grown for 12 h, 24 h, 48 h and 5 days (Supplementary Fig. 2) and the impact on YFP expression by PilB::Tn5/reporter was assessed. Note, conditioned media were supplemented with nutrients to negate possible nutrient limitation.

Individual flow cytometry experiments demonstrated a decrease in fluorescence intensity with CM age (e.g. Fig. 2b) in accordance with accumulation of the biofilm inhibitor during culture growth. Statistical analysis of three independent experiments indicated significant difference between rCV of cells grown in fresh medium and those grown in CM harvested at 12 h following culture initiation (12 h-CM; Supplementary Fig. 1b see section I of table). In addition, mean, median and rCV were significantly different between cells grown in fresh medium and those inoculated into CM harvested at 24 h, 48 h and 5 days (24 h-CM, 48 h-CM and 5d-CM; Supplementary Fig. 1b, section I of table). Moreover, mean and rCV were significantly different between cells exposed to 12 h-CM and those inoculated into either 24 h-CM, 48 h-CM or 5d-CM (Supplementary Fig. 1b, section II of table). Comparisons of the impact of CM from older cultures (Supplementary Fig. 1b, section III; 24 h-CM vs. 48 h-CM and 5d-CM and 48 h-CM vs. 5d-CM) did not reveal significant changes. Because individual experiments demonstrate accumulation of biofilm inhibitor in CM with growth time (e.g. Fig. 2b), we propose that the inhibitor is accumulated with culture age, which corresponds with culture density. Lack of significance, however, between data summarizing three biological repeats at 24 h or longer culture growth, indicates variable kinetics of inhibitor accumulation in independent experiments.

Interestingly, 12 h-CM had a significant impact on fluorescence rCV (Supplementary Fig. 1b) and apparently, less cells expressed the *ebfG*-operon at high levels compared with fresh medium (Fig. 2b). Yet, biofilm development by these cultures (Fig. 2d) suggests that the small fraction of *ebfG*-operon highly expressing cells is sufficient to drive biofilm formation. 24h-CM significantly affected the mean, median and rCV compared with fresh medium (Supplementary Fig. 1b, section I), however, in two of the three biological repeats biofilms were formed (Fig. 2d), in agreement with suggested variability in kinetics of accumulation of the biofilm inhibitor between individual experiments. 48h-CM and 5d-CM consistently inhibited biofilm formation in accordance with substantial repression of *ebfG*-operon expression under these conditions (Supplementary Fig. 1b section I and Fig. 2d). Together, data indicate presence of the inhibitor at early culture stages upon initiation of the logarithmic growth (Supplementary Fig. 2, Fig. 2b and Supplementary Fig. 1b), and suggest further accumulation with time and culture density.

### Localization of EbfG4

EbfG proteins do not share primary sequence similarity or domains with proteins of known function. To get insight into the role of these proteins in biofilm development, we selected EbfG4 for further characterization. This particular EbfG protein was chosen because a mutational approach impairing the secretion motif of either one of the EbfG proteins revealed that the mutation in EbfG4 had the most prominent impact on biofilm development compared with EbfG1-3^26^. To follow EbfG4 localization in biofilm-forming and planktonic strains we introduced a FLAG-epitope tagged EbfG4 (EbfG4::FLAG) to the double mutant having inactivations in both *pilB* and Synpcc7942_1134 (PilB::Tn5/EbfG4Ω). The double mutant grows planktonically—similarly to WT, 100% of the chlorophyll is in planktonic cells as assessed by measurement of the relative amount of chlorophyll in suspension of total chlorophyll in the culture (Supplementary Fig. 3). Insertion of a DNA fragment bearing either the native *ebfG*-operon or one encoding EbfG4::FLAG into the double mutant (PilB::Tn5/EbfG4Ω/Comp and PilB::Tn5/EbfG4Ω/EbfG4::FLAG, respectively), restored biofilm development; similarly to PilB::Tn5, <5% of the chlorophyll is in suspended cells (Supplementary Fig. 3). This analysis validated functionality of the tagged EbfG4.

**Fig. 3:**
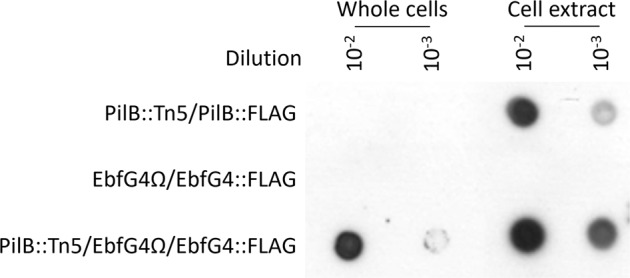
Dot-blot analysis using anti-FLAG antibodies. Whole cells and cell extracts of the following strains were analyzed: *pilB*-inactivated strain complemented with FLAG-tagged PilB (PilB::Tn5/PilB::FLAG), *ebfG4*-inactivated strain complemented with FLAG-tagged EbfG4 (EbfG4Ω/EbfG4::FLAG) and the latter strain that also harbors *pilB* inactivation (PilB::Tn5/EbfG4Ω/EbfG4::FLAG). Strains PilB::Tn5/PilB::FLAG and EbfG4Ω/EbfG4::FLAG are planktonic and PilB::Tn5/EbfG4Ω/EbfG4::FLAG forms biofilm. All data are derived from the same experiment and they were processed in parallel (see unprocessed image in supplementary information).

An initial indication that EbfG4 is localized to the cell surface was obtained by dot-blot analysis. Whole cells and cell extracts were spotted onto a nitrocellulose-membrane and probed with anti-FLAG antibodies (Fig. 3). This analysis suggested association of EbfG4 with the cell surface, as revealed by the signal obtained from whole cells (Fig. 3, PilB::Tn5/EbfG4Ω/EbfG4::FLAG). In contrast, the ATPase of T4P complex known to be localized cytoplasmically, was not detected in whole cells but only in cell extracts (Fig. 3, PilB::Tn5/PilB::FLAG), thus confirming availability of internal epitopes for detection only in cell extracts. EbfG4 was not detected in whole cells or in cell extracts of the planktonic strain EbfG4Ω/EbfG4::FLAG (Fig. 3). This result indicates that EbfG4 is neither secreted nor accumulated internally when the biofilm suppression mechanism is active, thus, we propose that under the growth conditions tested, the EbfG proteins do not have a role in WT cells.

**Fig. 4:**
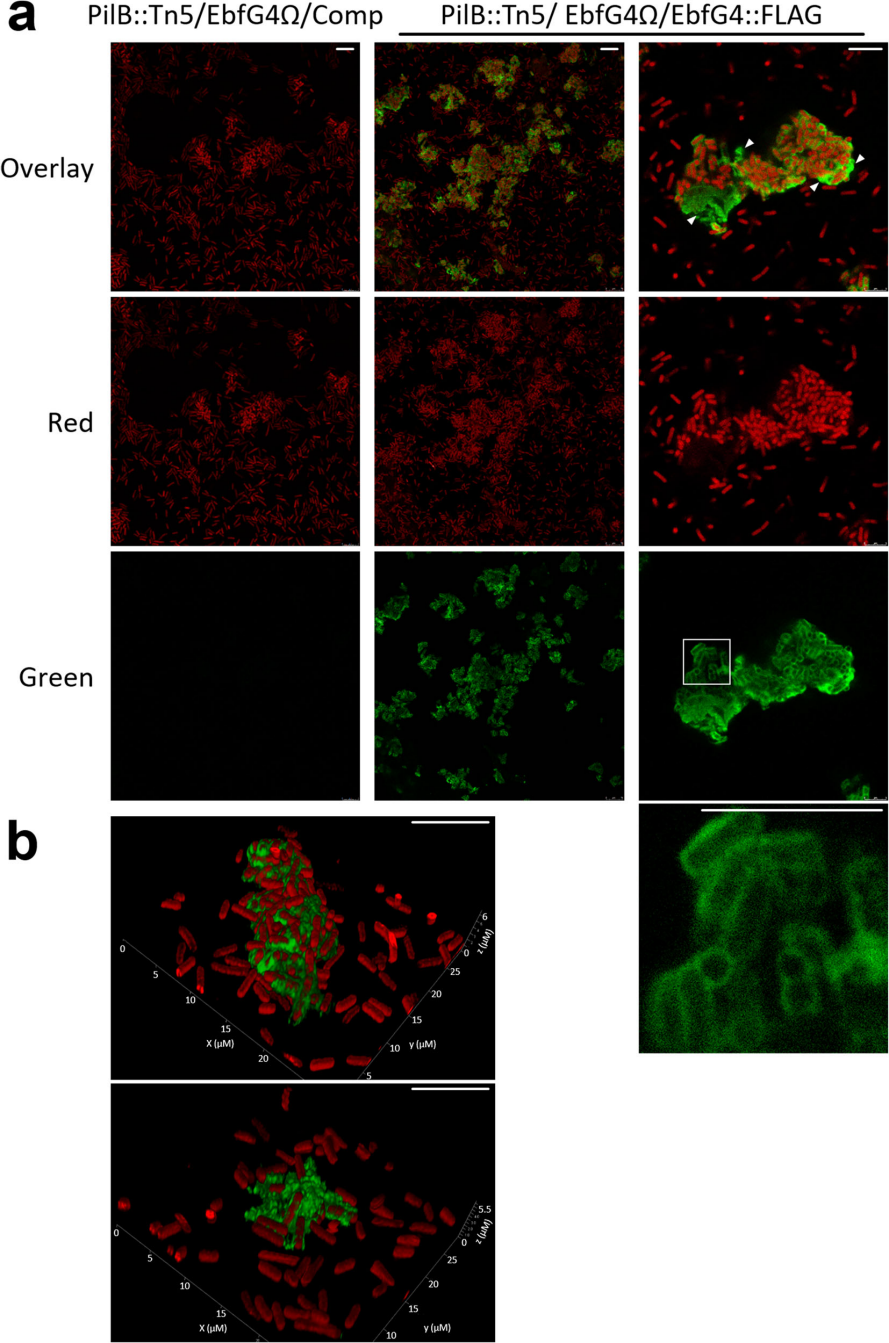
Visualization of EbfG4::FLAG in non-permeabilized cells by immunocytochemistry and confocal fluorescence microscopy. **a** Strains analyzed: PilB::Tn5/EbfG4Ω/Comp and PilB::Tn5/EbfG4Ω/EbfG4::FLAG. Red represents autofluorescence whereas green indicates the presence of EbfG4::FLAG. White square indicates the enlarged area shown in the panel below. Arrowheads point at patches of green fluorescence in areas void of cells. **b** 3D-imaging of strain PilB::Tn5/EbfG4Ω/EbfG4::FLAG. The scale bars correspond to 10 µm.

To follow up on the observation suggesting cell surface association of EbfG4 (Fig. 3), we examined by immunocytochemistry non-permeabilized cells that were subjected to anti-FLAG antibodies, thus allowing detection of only extracellular EbfG4. Green signal representing EbfG4 was detected only in strain PilB::Tn5/EbfG4Ω/EbfG4::FLAG but was absent from the cognate control strain PilB::Tn5/EbfG4Ω/Comp confirming specific detection of the FLAG epitope (Fig. 4a). Moreover, the green signal is associated with clustered cells (Fig. 4a, middle and right columns), while unclustered cells lack green signal and are visualized only by autofluorescence (Fig. 4a). Closer examination revealed green signal at the periphery of many of the clustered cells; therefore, we concluded that the EbfG4 protein is located at the surface of these cells (Fig. 4a, right column). In addition, patches of green fluorescence are observed in areas void of cells (Fig. 4a, right column, arrowheads), and 3D-imaging clearly indicates the presence of EbfG4 in between cells (Fig. 4b) supporting its role as a matrix protein.

**Fig. 5:**
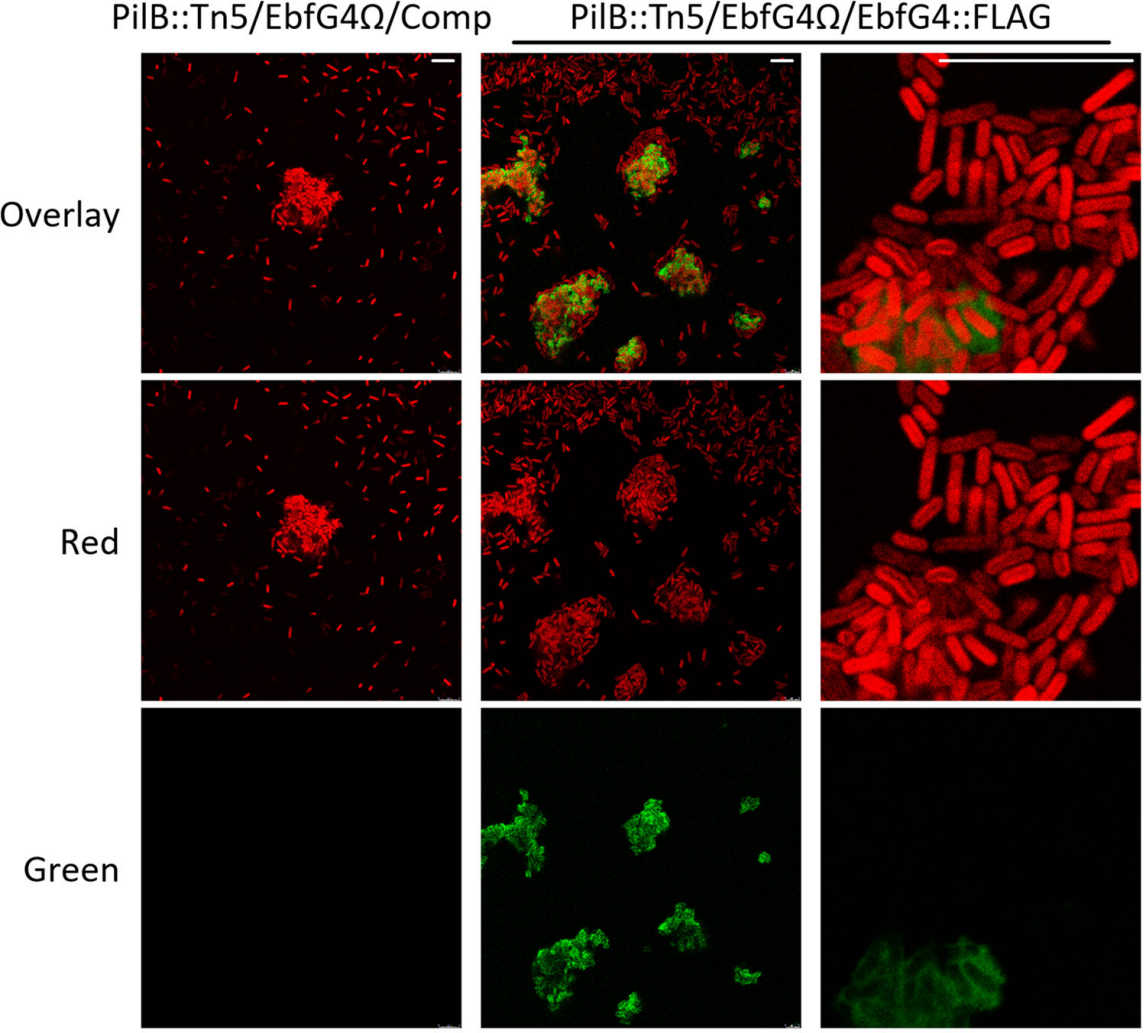
Visualization of EbfG4::FLAG in permeabilized cells by immunocytochemistry and confocal fluorescence microscopy. Strains analyzed: PilB::Tn5/EbfG4Ω/Comp and PilB::Tn5/EbfG4Ω/EbfG4::FLAG. Red represents autofluorescence whereas green indicates presence of EbfG4::FLAG. The scale bars correspond to 10 µm.

Next, we visualized permeabilized cells to test whether EbfG4 is also detected intracellularly. Images similar to those observed without permeabilization emerged from these analyses (Fig. 5). Close examination of an area mostly void of extracellular green fluorescence did not reveal green signal within the cells (Fig. 5, right column). Successful visualization of internal PilB::FLAG, exclusively in permeabilized cells, validate penetration of the antibodies used for detection (Supplementary Fig. 4). Together, these data indicate that EbfG4 does not accumulate intracellularly to detectable levels and suggest efficient secretion of this protein.

### Examination of amyloid formation by products of the *ebfG*-operon

**Fig. 6:**
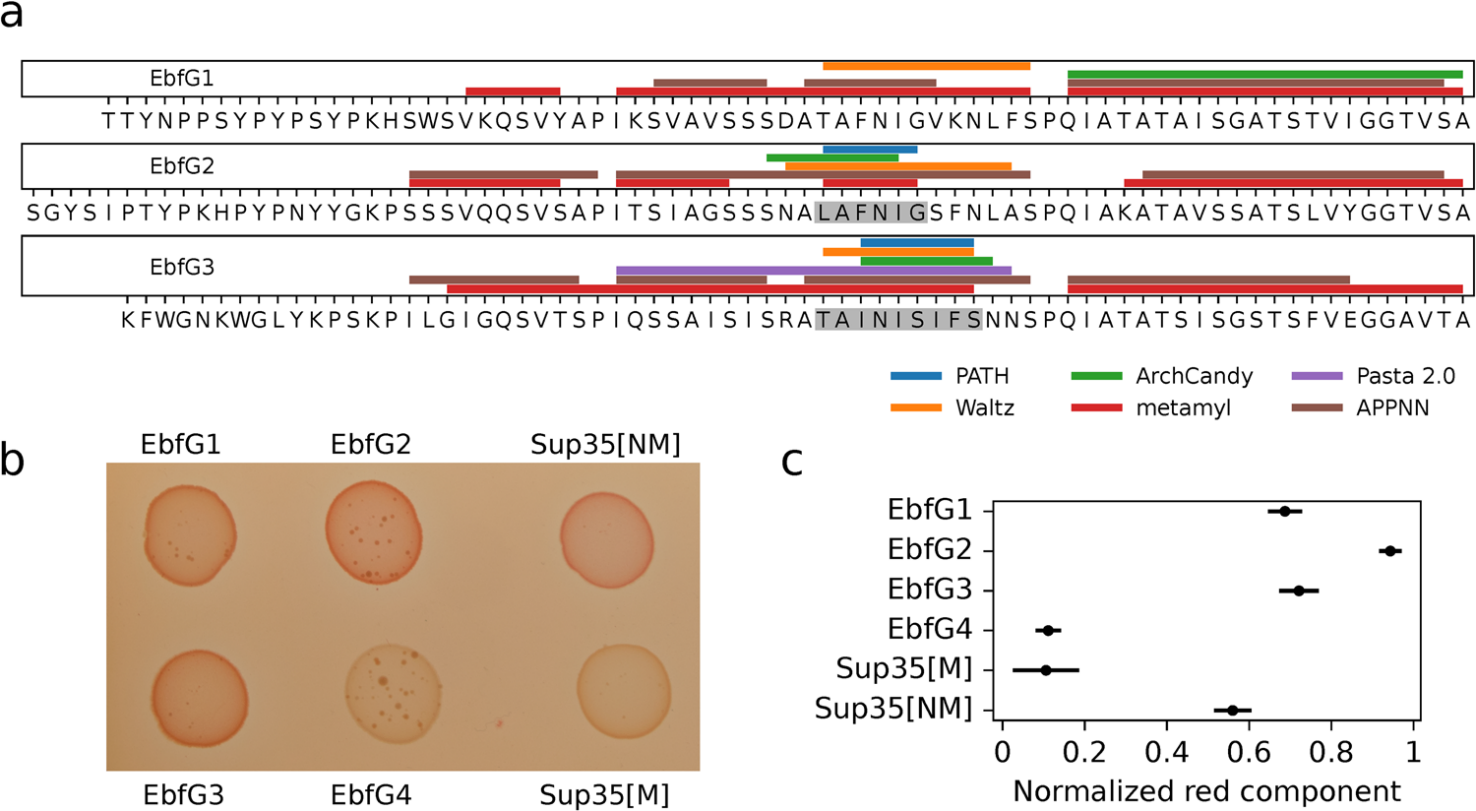
Amyloid prediction and heterologous expression of EbfG1-4 proteins. **a** Prediction of amyloidogenic hotspots in the sequences of EbfG1-3 using six software prediction tools. Shaded fragments correspond to consensus positive prediction in at least four of the predictors. **b** Colony color phenotype of EbfG-expressing *Escherichia coli* compared to negative (Sup35[M]) and positive (Sup35[NM]) controls for amyloid formation in inducing plates. **c** Normalized red component of the brim of each colony as analyzed in ImageJ, based on five biological replicates and displayed as the mean and 95% confidence interval (t distribution).

To test whether EbfG proteins might contribute to biofilm matrix formation through insoluble aggregates, we initially performed an in silico characterization of their tendency to fold as amyloids using a pipeline that consists of several prediction software to build consensus models. We found that EbfG2 and 3 had the highest prediction score, which was above the 0.5 cut-off for classification into amyloid-forming proteins. Using separate tools with diverse prediction methods for the identification of amyloid hotspots within the amino acid sequence, we found that the highly predicted regions occurred at the same location in the alignment of EbfG2 and EbfG3. Despite variations in the individual sequences, the core hotspot followed the motif AΦNIΠ, where Φ represents a hydrophobic residue, F or I, and Π, a small side chain residue, G or S (Fig. 6a). Such motif also occurs in EbfG1, however, in EbfG4 a polar uncharged amino acid (glutamine) is present instead of a hydrophobic residue (AQNIG). We modeled the amyloidogenic LAFNIG peptide from EbfG2 for its ability to form cross-beta structures. We found that the hexapeptide arranged in a steric zipper of antiparallel fashion (Supplementary Fig. 5), characteristic of amyloid proteins.

To support these predictions, we heterologously expressed the EbfG proteins to test for the formation of amyloids in vivo. To this end, we cloned the mature proteins in the Curli-Dependent Amyloid Generator (C-DAG) system fused to a 6x Histidine Tag at the C-terminus (Supplementary Table 1). After induction with arabinose, we found a phenotype for amyloid formation in EbfG1, 2 and 3, evident by the color shift of the colonies due to the binding of Congo Red, similarly to the positive control (Fig. 6b). Consistent with the in silico predictions, EbfG2 showed the strongest phenotype while EbfG4 showed no sign of amyloid formation, comparable to the negative control staining (Fig. 6c). Congo Red may also bind to exopolysaccharides. However, it is unlikely that C-DAG cells expressing different EbfG proteins vary in production and secretion of exopolysaccharides; thus, Congo Red staining in this system suggests binding to EbfG proteins. Indeed, further examination of amyloids using the specific stain AmyTracker supports the presence of amyloids for strains expressing EbfG1-3 (Supplementary Fig. 6), in agreement with their red colony phenotype in Congo Red-rich media (Fig. 6c).

**Fig. 7:**
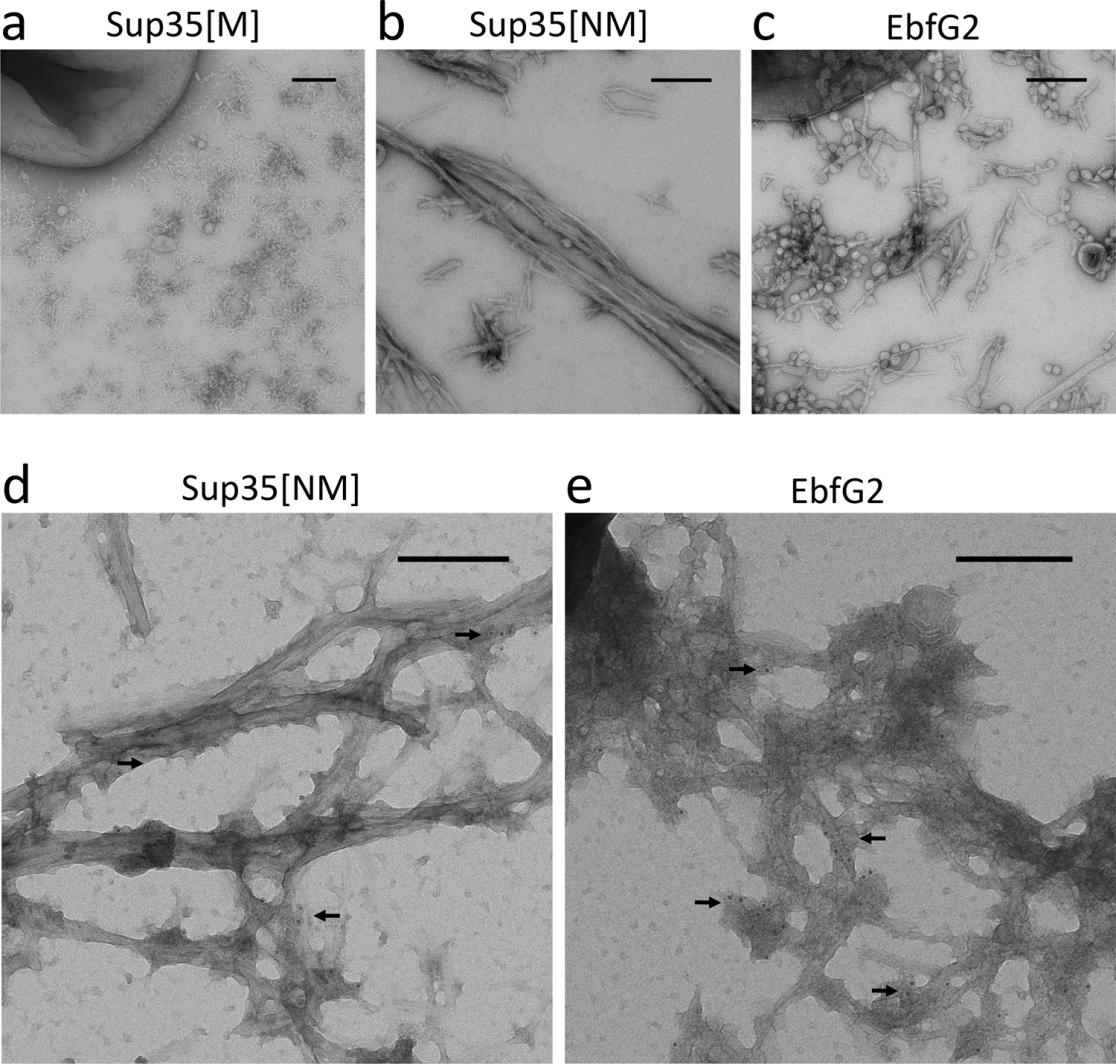
Transmission electron microscopy (negative staining) images of EbfG2 expressed in the C-DAG system. **a** Negative control (Sup35[M]) **b** Positive control (Sup35[NM]) **c** EbfG2 expressed in the C-DAG system. Fibril-like structures are found in the extracellular space for the positive control (Sup35[NM]) and EbfG2. **d**, **e** Immunogold stained samples where 6 nm gold marker particles localize to the fibril-like structures of the positive control (**d**) and EbfG2 (**e**). Arrows indicate gold particles. The scale bars correspond to 200 nm.

When looking at the induced EbfG2-producing C-DAG cells under the transmission electron microscope (TEM), we detected fibril-like structures in the extracellular space (Fig. 7c). The fibrils resemble those produced by the positive control (Fig. 7b), however shorter in length and associated with vesicle-like structures (Fig. 7c). Using immunostaining methods, we corroborated the identity of the fibrils as containing EbfG2 protein, based on the colocalization of gold nano-beads directed to the 6x Histidine tag from the protein. We observed unevenness in the distribution of the labeling which could be due to variable antibody accessibility of the tag, both for the positive control and EbfG2 (Fig. 7d, e).

## Discussion

Previous genetic and point mutation analyses demonstrated the requirement of the EbfG proteins for biofilm formation^23,26^. As manifested by analysis in individual cells, expression of the operon encoding these genes varied substantially more in the biofilm-forming mutant PilB::Tn5 than among WT cells (Fig. 2a, b and Supplementary Fig. 1). Although ~75% of PilB::Tn5 cells express the *ebfG*-operon at similar or lower level compared to WT (Fig. 2c), ~90% of the cells of this strain are found in the biofilm (Supplementary Fig. 3^23,26,28^). These findings are consistent with cell specialization during *S. elongatus* biofilm development. Given that EbfG1-3 are prone to form amyloids (Fig. 6) and TEM analysis revealed that EbfG2 forms fibrillar structures similar to amyloids, we propose that a relatively small subpopulation of PilB::Tn5 culture produces matrix components that support robust biofilm development by the majority of the cells. Taken together, we propose cell specialization in production of so-called public goods. Such a phenomenon may confer selective advantage because only a minority of the cells invest resources for the benefit of the population, which gains protection within the biofilm^29^. Cell specialization in microbes have been documented in heterotrophic bacteria, for example, during matrix formation in *Bacillus subtilis*^30^. Such differentiation, however, was not previously reported for cyanobacterial biofilm development, thus, this study, which uncovers cell differentiation suggests division of labor in communities of these ecologically important photosynthetic prokaryotes.

Comparisons of expression of the *ebfG*-operon in fresh medium and under CM harvested at different time points along growth of WT culture indicate production and secretion of the inhibitor at early culture stages and suggest further accumulation with time and cell density. The amount of biofilm inhibitor present following 12 h growth is sufficient to affect reporter expression (Fig. 2b and Supplementary Fig. 1b), yet is insufficient for biofilm inhibition (Fig. 2d). Up to 24 h the inhibitor gradually accumulates (Supplementary Fig. 1b) and at 48 h the inhibitor reaches levels that consistently inhibit biofilm development (Fig. 2d). Together, data imply a density-dependent mechanism, however, a gradual impact of the inhibitor is observed rather than a “threshold-like” effect typical of quorum-sensing mechanisms known in heterotrophic bacteria, e.g., induction of the lux-operon^31–34^. Cyanobacterial quorum-sensing is largely unknown, although N-octanoyl homoserine lactone was suggested to be involved in quorum-sensing in *Gloeothece* PCC6909^35^. Biosynthetic LuxI-like proteins, which are responsible for production of acylated homoserine lactones in numerous heterotrophs, however, are not encoded in the majority of cyanobacterial genomes^36^, therefore, such molecules are not likely to represent a general mechanism for cyanobacterial intercellular communication. An additional study demonstrated a governing role of extracellular signals produced at high density on transcription of particular genes in low-density cultures of *N. punctiforme* PCC 73102. These data support the existence of a quorum-sensing-like mechanism(s), however, the nature of the signal(s) and the regulatory network have yet to be identified^36^.

Microscopic analysis revealed that some of the cells of PilB::Tn5/EbfG4Ω/EbfG4::FLAG present the EbfG4 protein on their cell surface (Figs. 4a and 5). Interestingly, EbfG4 is only observed on the surface of clustered cells and cells that lack EbfG4 labeling are dissociated from the clusters (Figs. 4a and 5). Together, these observations are in accordance with an adhesion function of EbfG4. In addition, EbfG4 was observed in the intercellular space (Figs. 4 and 5), consistent with the hypothesis that it serves as a matrix component. It is possible that similarly to the adhesin protein SasG of *Staphylococcus aureus*^37^, EbfG4 is initially deposited to the cell surface and later is shed to the extracellular matrix. The role of EbfG4 in the matrix is unknown, however, although it does not form amyloids by itself (Fig. 6) it may be associated with amyloid structures formed by EbfG1-3.

When establishing biofilms, microbes require a resilient scaffold on which the cells can settle. Bacteria from diverse ecosystems have solved this problem by producing and releasing functional amyloids into their environment^38,39^. Amyloid proteins are able to assemble into long and strong fibrils, which can withstand chemical and physical stresses^40^. However, the production of amyloids is a process that can easily get out of control, therefore, it requires a complex and dedicated machinery for appropriate manufacturing. Here, we have investigated the amyloid forming capabilities of the *ebfG*-operon proteins and found strong evidence supporting amyloid formation in EbfG1-3, and most manifestly in EbfG2. Modeling of the amyloid hotspot peptide within EbfG2 revealed arrangement in a steric zipper of antiparallel fashion (Supplementary Fig. 5), characteristic of amyloid proteins^41^. EbfG4, which has a prominent role in biofilm formation, however, did not spontaneously form amyloid fibrils. Consistent with homology in the amyloid hotspots, we hypothesize this could be related to a mechanism intended to control aggregation. By separating the amyloid nucleators, in this case EbfG1-3, from other components of the fibril, e.g. EbfG4, better control over the synthesis of amyloids could be achieved. This would be analogous to, for example, the functioning of CsgB and CsgA in the production of curli, the biofilm backbone in *E. coli*^42^. As observed in the TEM pictures, the EbfG2 fibrils were much shorter than the positive control and did not bundle together. Heterogeneous fibrils formed of several EbfG proteins could result in more stable fibrils, as seeding of amyloids composed of perfect repeats has been shown to cause fragmentation^43^.

Given the amyloid nature of EbfG1-3 proteins one may speculate that these matrix components of *S. elongatus* biofilms assist in recruitment of additional cells of this cyanobacterium or of other microbes for establishment of multispecies biofilms. Recruitment of cells that have not yet initiated the synthesis of exopolysaccharides by a proteolysis product of the matrix protein RmbA was demonstrated in *Vibrio cholerae*^44^.

Our findings pave the way for controlling formation of unwanted biomats, by using amyloid disrupting compounds, as already shown for other bacteria^45,46^. On the other hand, intentional use of protein seeds could facilitate stronger amyloids and hence elicit formation of beneficial biofilms.

## Methods

### Strains and culture conditions, biofilm assay, and harvesting of conditioned medium (CM)

*S. elongatus* PCC 7942, an obligatory photoautotroph, and all derived strains were grown in mineral medium BG11, prepared from stock solutions as follows: Stock I (100× concentrated, autoclave): NaNO_3_—150.00 g/L, MgSO_4_·7H_2_O—6.50 g/L, CaCl_2_·2H_2_O—3.60 g/L; Stock II (100× concentrated, autoclave): K_2_HPO_4_—3.05 g/L, Na_2_Mg EDTA—0.10 g/L; Stock III (100× concentrated, prepare fresh, no need to sterilize), C_6_H_11_FeNO_7_—0.60 g/L, C_6_H_8_O_7_—0.60 g/L; Stock V (1000× concentrated, sterilize by 0.22 μm filter), H_3_BO_3_— 2.86 g/L, MnCl_2_·4H_2_O—1.84 g/L, ZnSO_4_·7H_2_O—0.22 g/L, NaMoO_4_·2H_2_O—0.39 g/L, CuSO_4_·5H_2_O—0.08 g/L, Co(NO_3_)_2_·6H_2_O—0.05 g/L; HEPES buffer—119.15 g/L (25× concentrated) titrated to pH 8.0 with 10 M NaOH and autoclave. Dilute stock solutions in double distilled water and autoclave. Cultures were grown at 30 °C in Pyrex tubes under bubbling with air enriched with CO_2_ (see supplementary video). Details of infrastructure for bubbling are provided in refs. ^47,48^. Incandescent light was provided at flux of ~30 μmol photons m^−2^ s^−1^. Construction of mutants and details of molecular manipulations are provided in Supplementary Table 1.

For biofilm assessment, planktonic cells were removed from the sessile fraction. Quantification is based on chlorophyll measurement as a proxy for biomass accumulation in sessile as well as in planktonic cells and representation of the relative fraction of chlorophyll in planktonic cells. Chlorophyll was extracted in 80% acetone and quantified based on absorbance at OD_663_^47^.

For harvesting of CM, WT cultures were initiated from liquid starters at OD_750_ = 0.2. For collection of CM, cultures were centrifuged (5000 × *g*, 10 min) at room temperature, and the supernatant was removed and passed through 0.22 µm filter. This CM was supplemented with nutrients by addition of medium stock solutions as in the preparation of fresh growth medium.

### Flow cytometry

50 ml culture at exponential phase was centrifuged (6000 g, room temperature), resuspended with 4 ml fresh BG11 to obtain a concentrated culture for inoculation into fresh medium or CM at an OD_750_ of 0.5. Aliquots of 0.5 ml were taken from each culture tube following 6 days of growth and then, in case of biofilm-forming strains, planktonic cells were removed. 1.5 ml BG11 were used to resuspend the biofilmed cells by rigorous pipettation and 0.13 ml were transferred to a 1.5 ml Eppendorf tube for homogenization with a pellet pestle (Sigma-Aldrich, Z359971-1EA). The homogenized samples were filtered through a mesh (pore size 52 µm), supplemented with formaldehyde to a final concentration of 1%, diluted with phosphate-buffered saline (PBS) to OD_750_ of ~0.0001 and measured using BD *FACSAria* (excitation 488 nm, emission 530 ± 30 nm). Gating for flow cytometry analysis was based on cyanobacterial autofluorescence (Supplementary Fig. 7).

All statistical analyses were conducted in the statistical program R, version 3.3.2^49^. FCS files obtained from *FlowJo* were analyzed with *flowcore* package^50^. Mean, median, and robust coefficient of variation (CV) of the intensity distribution for each sample were calculated. Robust CV was calculated as defined in the *FlowJo* documentation https://docs.flowjo.com/flowjo/workspaces-and-samples/ws-statistics/ws-statdefinitions/. Intensity values were log-transformed. Significant difference between biofilm and planktonic cells of a particular culture was tested using Paired t-tests on several intensity distribution parameters (mean, median and robust CV). Initial analysis did not reveal significant differences between biofilm and planktonic cells within a particular culture, therefore, these data were combined for further analysis. Effect of growth medium or genetic background on intensity distribution parameters (mean, median and robust CV) was tested with two-way repeated measures ANOVA. Specifically, mixed linear effect models were fitted with medium or genetic background as fixed effects and biological replicates as random effect, (using *lmerTest* package^51^), and the ANOVA was performed on the resulting models. Post hoc pairwise comparisons were performed by testing linear contrasts (using emmeans R package^52^), and FDR correction was applied to control for multiple testing. Normality of residuals and homogeneity of variances assumptions were checked graphically.

### Dot-blot analysis

For preparation of cell extracts, 50 ml 6 days old cultures were concentrated by centrifugation (5000 × *g*, 10 min), resuspended with 0.5 ml TE (10 mM Tris-HCl pH 8., 1 mM EDTA) and freshly prepared protease inhibitor cocktail (Sigma, P8465-5ML) was added to 0.86 mg/ml. Glass beads (0.2 g) were added for breakage by mixer mill (Retch MM400) at a frequency of 30 s^−1^ for 2 min in pre-chilled holders (5 times, 1 min incubation on ice between the cycles). Cell lysates were centrifuged (835 × *g*, 5 min, 4 °C) to pellet the glass beads. Cell lysates were transferred to a fresh Eppendorf tube, diluted with TE supplemented with protease inhibitor cocktail and 2.3 µl from diluted extracts were spotted onto TransBlot Turbo nitrocellulose membrane (Bio-Rad) and air dried for 5 min. All following procedures were performed at room temperature: Blocking was done for 1 h in 0.1% bovine serum albumin in TBST (20 mM Tris-HCl (pH 8.0) and 0.05% Tween20). Incubation with anti-FLAG (ab1162, Abcam; 1:2000 diluted in blocking solution) was performed for 1 h following three washes in TBST for 5 min each. Incubation with secondary antibodies (goat anti rabbit IgG, 170-6515, Bio-Rad; 1:5000 diluted in blocking solution) was done for 1 h following washes as above with extension of last wash to 15 min and signal detection using SuperSignal West Pico kit (Thermo Scientific, 34080).

### Fluorescence microscopy

Cultures were initiated and grown as described for biofilm quantification. Cultures (30 ml) were centrifuged (5 min, 6000 × *g*, room temp) and resuspended in 1 ml PBS. In case of biofilm-forming strains, the planktonic cells were removed with a pipette and the biofilmed cells were gently scraped and resuspended using 1 ml PBS. Rigorous pipetting was avoided to preserve clusters for microscopy analyses. The concentrated cultures were precipitated by centrifugation in Eppendorf tubes as above, resuspended in 1 ml PBS and formaldehyde, from 16% stock solution prepared as described in Cold Spring Harbor Protocols (http://cshprotocols.cshlp.org/content/2010/1/pdb.rec12102.full), was added to a final concentration of 2%. Cells were incubated in the dark (30 min at room temperature in a tube rotator followed by 30 min incubation on ice), washed once in PBS, resuspended in 1 ml PBS and the mixture was equally divided into two Eppendorf tubes. These tubes were centrifuged—cells for imaging without permeabilization were resuspended in 1 ml PBS and saved in the dark at room temperature. For imaging following permeabilization, cells were resuspended in 500 µl 0.1% triton in PBS, incubated at room temperature in a tube-rotator for 15 min and centrifuged. Cell pellet was resuspended with lysozyme solution (0.2 mg/ml dissolved in 50 mM Tris-HCl, pH 7.5 and 10 mM EDTA) and incubated for 30 min at 37 °C. Such lysozyme treatment allowed penetration of antibodies while preserving cell morphology. Cells were washed twice with 1 ml PBS. An aliquot of 200 µl was treated with an equal volume of freshly prepared blocking solution (5% BSA in PBS) in a tube-rotator for 1 h at room temperature. Cells were pelleted, resuspended in 100 µl anti-FLAG antibody (Abcam, 1:400 diluted with blocking solution), incubated for 40 min at room temperature and then 40 min at 30 °C. Cells were washed twice with 100–200 µl PBS buffer, resuspended in 20 µl secondary antibody (Alexa Fluor® 488 Abcam) diluted 1:100 in blocking solution and incubated for 1 h at 30 °C. Pellet was washed once with 50 µl PBS and resuspended in 20 µl PBS. 3–5 µl were spread on microscopy slides prepared as follows. 10 µl of L-polylysine (Sigma) diluted 1:10 was spread on a microscope slide (approximately on a 1 cm × 1 cm region). Slides were air dried, washed by dipping them twice in double distilled water and air dried. Cells were layered on the coated area, air dried and slides were centrifuged in 50 ml falcon tubes to attach cells to the polylysine layer (300 × *g* 10 min, room temperature). 3 µl antifade^53^ was spotted and covered with a coverslip. Images were recruited using Leica SP8 confocal microscope, driven by the Lasx acquisition software v.3.5.5 (Manheim, Germany). The Leica white light laser (WLL) was set at 50% max power. The native autofluorescence that was used to image the cells (red channel) was excited with the WLL set at 630 nm (1% slider), and the emission was collected between 640 and 720 nm. The green channel (goat anti-rabbit Alexa Fluor 488 secondary antibody (Abcam)), was excited at 488 nm (2% slider), and the emission collected between 505 and 550 nm, using a HyD detector with time gating activated, between 0.1 and 10 ns, to further lower autofluorescence.

The overview images were acquired with a Leica HC PL APO CS2 63×/1.40 oil objective, at a resolution of 120 nm/pixel (0.75× zoom), with 5× averaging. High magnification images were acquired with a Leica HC PL APO CS2 100×/1.40 oil objective at a resolution of 28 nm/pixel (4× zoom). Between 70 and 90 Z-slices were acquired of each field, at 150 nm intervals. Z stacks were exported from LASX directly to Huygens (SVI, Netherlands), deconvolved with the default settings (30 iterations), imported back into LASX, collapsed to a 2D image with the built-in LasX “maximum projection” algorithm, and exported as TIF files.

### Amyloid analysis

We employed the TANGO algorithm and the machine learning programs APPNN and AmyloGram for in silico amyloid prediction over the mature peptide sequence of EbfG1-4^54–56^. The pipeline can be found at https://github.com/danielzmbp/amypred. After max-min normalization of the scores between 0 and 1, the cutoff for amyloid prediction was set at 0.5. For the annotation of amyloidogenic hotspots, we employed software with a diverse predictive background, including statistical sequence analysis (WALTZ), structural information analysis (ArchCandy and Pasta 2.0), machine-learning-based (APPNN and PATH), and metamyl, a consensus predictor^56–61^. We predicted the cross-beta three-dimensional structure from the amyloid peptide domains using Cordax and visualized it using ChimeraX^62,63^.

We employed the Curli-Dependent Amyloid Generator (C-DAG) system to study amyloid formation in vivo^64,65^. This system uses the built-in curli processing system from *Escherichia coli* to express recombinant proteins in order to test for their amyloid aggregation. Positive and negative controls for amyloid formation employed, the *Saccharomyces cerevisiae* prion Sup35 with aggregating domain (Sup35[NM]) and without (Sup35[M]), were encoded by pVS72 and pVS105 plasmids, respectively. EbfG proteins equipped with a CsgA secretion signal in place of the native secretion signal and fused to a 6x Histidine tag at the C-terminus were separately cloned in pExport plasmids and expressed in the *E. coli* strain VS45 (Supplementary Table 1). For colony color phenotype analysis in inducing Congo Red plates (LB agar, 100 mg/l carbenicillin, 25 mg/l chloramphenicol, 0.2% w/v L-arabinose, 1 mM IPTG and 10 mg/l Congo Red), colonies were grown for four days at 22 °C in the dark.

To add support for the formation of amyloids of the candidate EbfG proteins, we stained C-DAG cultures grown for four days on inducing plates lacking Congo Red with the amyloid-specific AmyTracker™ 680 red dye. The cells were resuspended in PBS and adjusted to an optical density at 600 nm of 6. After incubation with AmyTracker (1 µg/ml final concentration) and shaking for 30 min at room temperature, samples were visualized on an inverse LSM880 confocal microscope (Zeiss). Excitation was achieved with the 561 nm laser line and resulting emission wavelengths between 600 and 750 nm visualized.

For transmission electron microscopy (TEM) analysis, we deposited cells grown for four days in inducing Congo Red plates on plastic and carbon-coated copper mesh grids. After drying, we incubated with anti-polyhistidine primary antibody for 1 h (mouse peroxidase-coupled IgG2, 1:200, Sigma Aldrich A7058) followed by incubation with secondary anti-mouse IgG conjugated to 6 nm gold beads for 1 h (goat, 1:30, Dianova) for the immunostained samples. All samples were negatively stained for 30 s in aqueous uranyl acetate, before visualization in a JEM 1400 Plus transmission electron microscope (JEOL). Controls on strains not producing fibrils and omitting primary antibodies were negative for background staining.

### Reporting summary

Further information on research design is available in the Nature Research Reporting Summary linked to this article.

## Supplementary information

Supplementary Information

Reporting Summary

Supplementary video

## Data Availability

The data underlying this article are available within the article and the accompanying supplementary information. Additional data are available from the corresponding author upon request.
