# Supplementary material for: Cell specialization in cyanobacterial biofilm development revealed by expression of a cell-surface and extracellular matrix protein
Source: NPJ Biofilms Microbiomes. 2023 Mar 2;9:10. doi: 10.1038/s41522-023-00376-6 (PMC9981879; doi:10.1038/s41522-023-00376-6)
Supplement: Supplementary file 1 — Supplementary Information [file 41522_2023_376_MOESM1_ESM.pdf]

Supplementary Information

a

Strains compared		Mean	Median	Robust CV
WT/reporter	PilB::Tn5/reporter	t(38)=0.5934 p=0.6624	t(38)=1.2514 p=0.364	t(38)=-12.323 p<0.0001
	PilB::Tn5	t(38)=16.8162 p<0.0001	t(38)=17.0292 p<0.0001	t(38)=-9.7165 p<0.0001
	WT	t(38)=15.671 p<0.0001	t(38)=15.8606 p<0.0001	t(38)=-7.583 p<0.0001
PilB::Tn5/reporter	PilB::Tn5	t(38)=16.2228 p<0.0001	t(38)=15.7778 p<0.0001	t(38)=2.6064 p=0.0314
	WT	t(38)=15.0775 p<0.0001	t(38)=14.6092 p<0.0001	t(38)=4.74 p=0.0001
PilB::Tn5	WT	t(38)=-1.1453, p=0.4301	t(38)=-1.1686, p=0.3896	t(38)=2.1336, p=0.0862

b

	Growth Medium compared		Mean	Median	Robust CV
Section I	Fresh Medium	12h-CM	t(38)=-1.3025 p=0.3521	t(38)=-1.7122 p=0.2015	t(38)=-3.6798 p=0.0022
		24h-CM	t(38)=-3.9282 p=0.0009	t(38)=-3.9339 p=0.001	t(38)=-6.4814 p<0.0001
		48h-CM	t(38)=-5.264 p<0.0001	t(38)=-5.2079 p<0.0001	t(38)=-8.4909 p<0.0001
		5d-CM	t(38)=-5.1203 p<0.0001	t(38)=-5.1009 p<0.0001	t(38)=-8.7955 p<0.0001
Section II	12h-CM	24h-CM	t(38)=2.6258 p=0.0289	t(38)=2.2217 p=0.0755	t(38)=2.8016 p=0.0214
		48h-CM	t(38)=3.9615 p=0.0009	t(38)=3.4957 p=0.0032	t(38)=4.8111 p=0.0001
		5d-CM	t(38)=3.8178 p=0.0013	t(38)=3.3887 p=0.0041	t(38)=5.1157 p<0.0001
Section III	24h-CM	48h-CM	t(38)=1.3358 p=0.3492	t(38)=1.274 p=0.3592	t(38)=2.0095 p=0.1095
		5d-CM	t(38)=1.192 p=0.4108	t(38)=1.167 p=0.3896	t(38)=2.3141 p=0.0591
	48h-CM	5d-CM	t(38)=-0.1437 p=0.9126	t(38)=-0.107 p=0.9564	t(38)=0.3046 p=0.8748

p ≤ 0.05
p ≤ 0.005
p ≤ 0.0005

Supplementary figure 1: Summary of statistical analyses of reporter expression. Strains analyzed: WT, PilB::Tn5 and their cognate reporter strains grown in fresh medium **(a)** and PilB::Tn5/reporter cells **(b)** grown in fresh medium or conditioned medium (CM) . Results are reported in the following form: t(df) = t-value, p = adjusted p-value. Data were calculated from three independent repetitions.

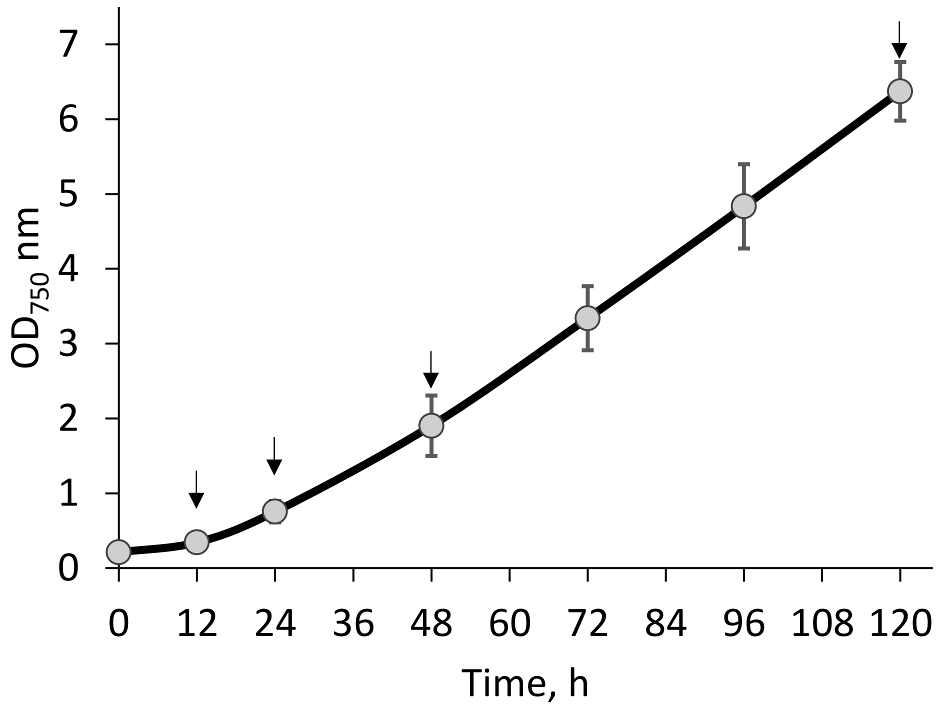

Supplementary figure 2: Growth of WT cultures as measured by OD_{750} nm as a function of time. Data represent averages and standard deviations from three biological repeats. Arrows indicate time points at which CM was harvested.

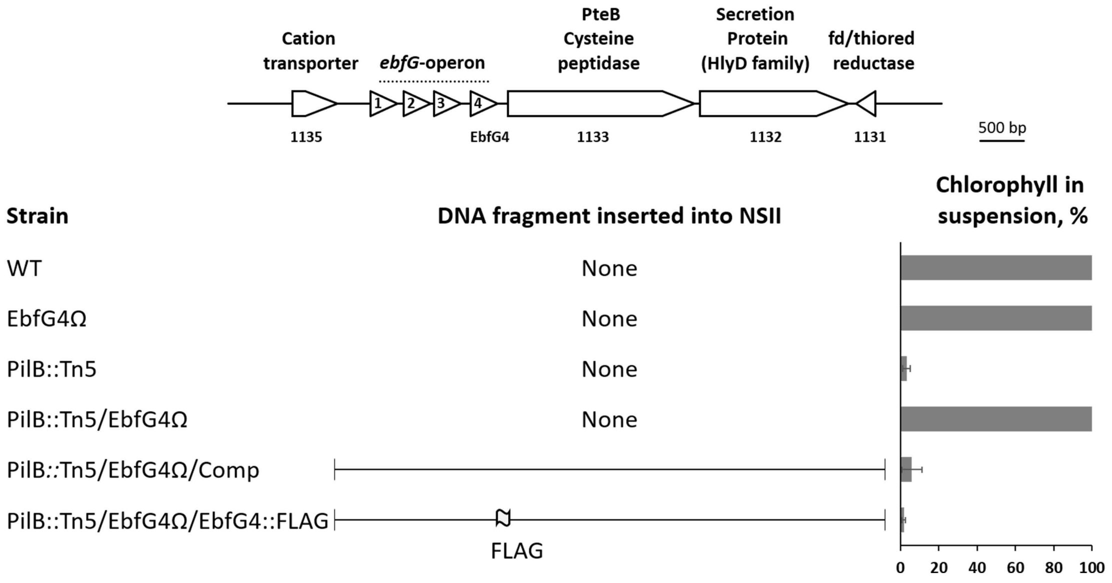

Supplementary figure 3: FLAG-tagged EbfG4 is functional in biofilm development.

Genomic region of the *ebfG*-operon. Bar graph presents percentage of total chlorophyll in the suspended cells (average of three independent biological repeats ± standard deviation). Strains analyzed include: WT, a strain in which *ebfG*4 was insertionally inactivated (EbfG4Ω), PilB::Tn5, the double mutant PilB::Tn5/EbfG4Ω, double mutants complemented with the indicated fragments encoding native or FLAG-tagged EbfG4 (PilB::Tn5/EbfG4Ω/Comp and PilB::Tn5/EbfG4Ω/EbfG4::FLAG, respectively.

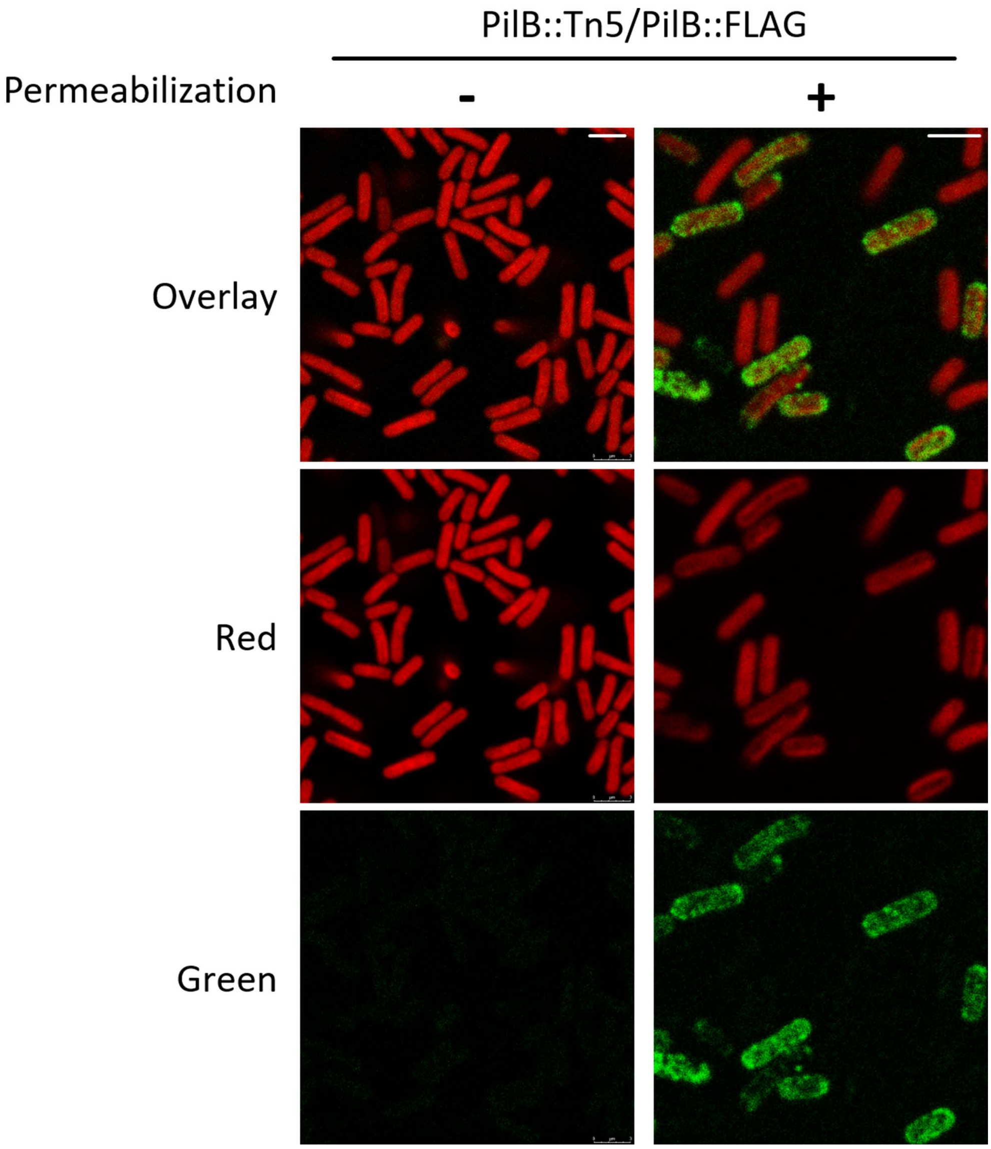

Supplementary figure 4: Immunocytochemistry with or without permeabilization of strain PilB::Tn5/PilB::FLAG.

Red represents autofluorescence whereas green indicates presence of EbfG4::FLAG. The scale bars correspond to 3 µm.

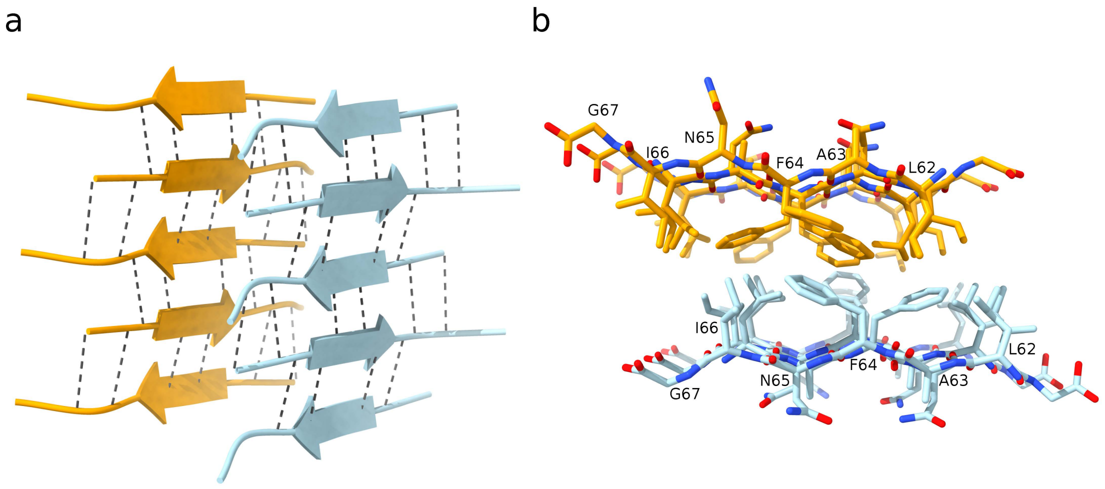

Supplementary figure 5: Cross-beta structure modelling of amyloidogenic LAFNIG peptide from EbfG2.

a Three-dimensional model of the LAFNIG peptide displaying a cross-beta antiparallel structure. In dashed black lines, hydrogen bonds holding together each cross-beta sheet are depicted. **b** Upper view of the structure highlighting the steric zipper, where hydrophobic residues (L, A, F and I) are concentrated in between the beta-sheets and hydrophilic residues (N and G) directed towards the outside.

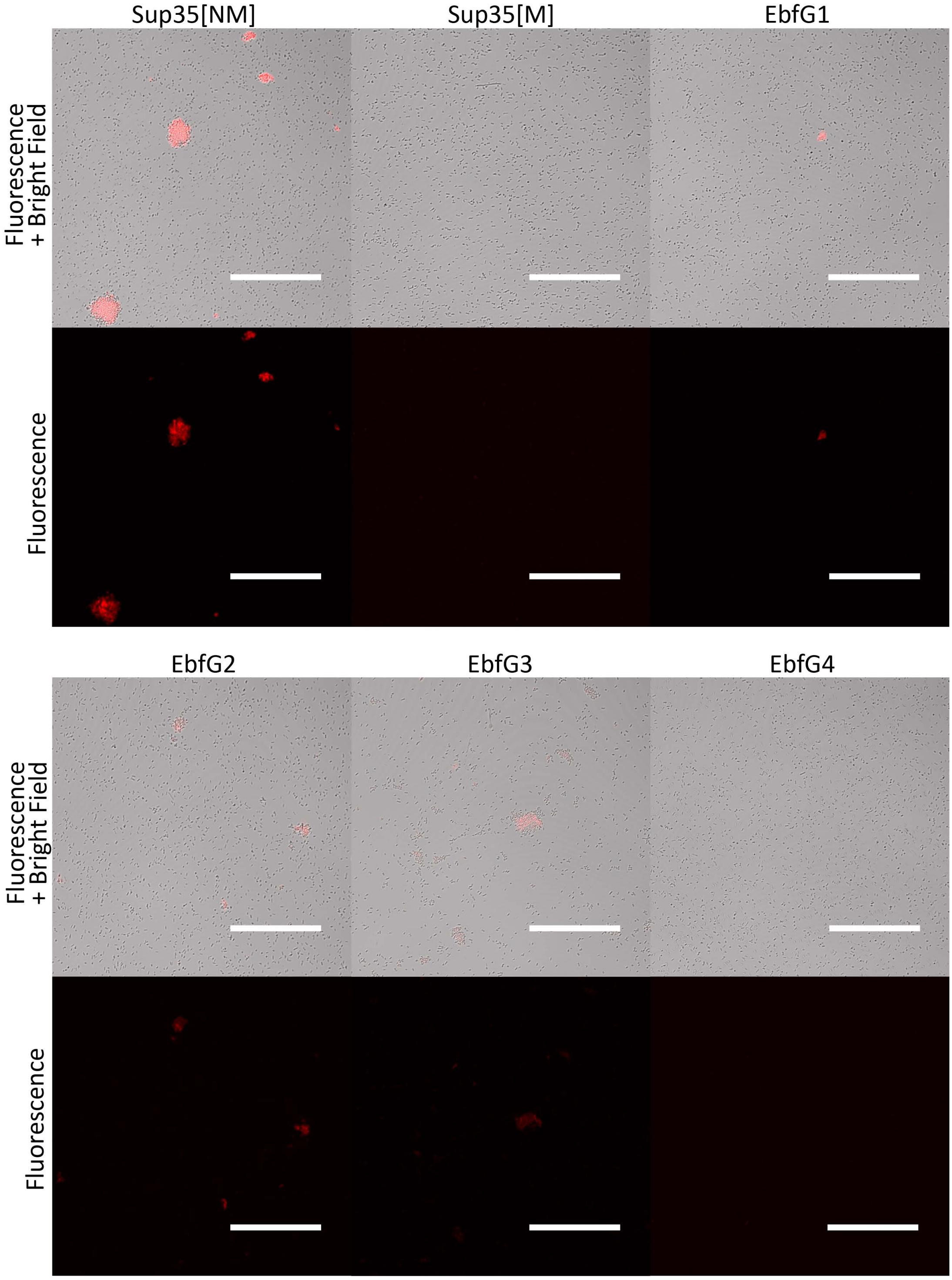

Supplementary figure 6: AmyTracker 680 staining of *Escherichia coli* cultures expressing EbfG proteins.

For each strain, the isolated fluorescence emission (600-750 nm) and the brightfield plus fluorescence overlays are depicted. Also shown are positive controls for amyloid formation, Sup35[NM], and no amyloid formation, Sup35[M]. The scale bars correspond to 50 µm.

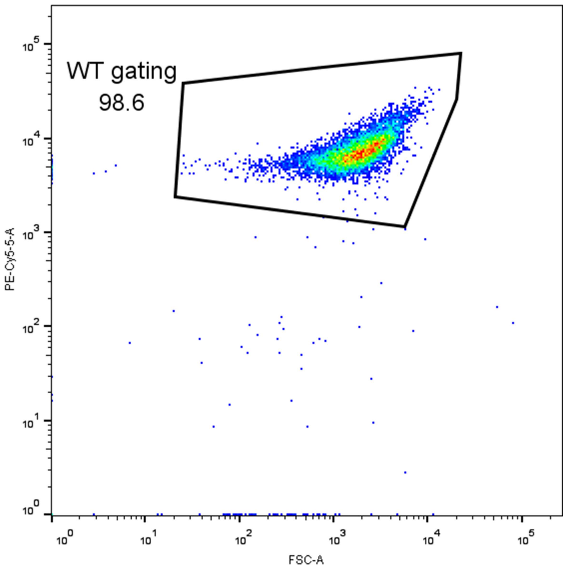

Name	Statistic	#Cells
060120 #310_001_310_001.fcs		10191
**WT gating**	98.6	10045

Supplementary figure 7: Gating for flow cytometry analysis based on cyanobacterial autofluorescence.

**Cloning of FLAG-tagged EbfG4[1]**		
**Primer Name**	**Primer sequence (upper forward, lower reverse)**	**Purpose**
RP10-swaI-F	CGGCCAATAACCCAGGGATTTTGTACTGCGACTCGACCAAG	*Cloning FLAG Fragment #1*
RP10-swaI-R	CTGCCGGGGAGCTCCTTCATTTGTTATGAACGTTGGCGATCG	*Cloning Fragment #3*
EbfG4-FLAG-R	CTTATCGTCGTCATCCTTGTAATCAAGATTGACAGCTACGTTAATGG	*Cloning FLAG Fragment #1*
EbfG4-FLAG-F	GATTACAAGGATGACGACGATAAGTAGCCAGTGCGATCGCAAG	*Cloning FLAG Fragment #2*
NS2-2F	CTCTTGGTGCTGTTCAGTC	*Sequencing*
**Cloning of YFP-reporter [2]**		
**Primer name**	**Primer sequence (upper forward, lower reverse)**	**Purpose**
P-EbfG::YFP Forw	AGTCGGCCAATAACCCAGGGATTTTGTACTGCGACTCGACCAAG	P-EbfG::YFP, *Fragment #1*
P-EbfG_RBS::YFP tail Rev	CACCATCTTAAGACCTCCTTTATTTTTTTTGATTCGCCCTTGACTC	
P-EbfG:: YFP_Forw	AGTCGGCCAATAACCCAGGGATTTTGTACTGCGACTCGACCAAG	P-EbfG::YFP, *Fragment #2*
YFP_genomic 3’ UTR Rev	CATCCGTCAGGATGGCCTTCTCCTGCAGGGCCGAGTTTGTACAAGAAAGC	
NSI- pRP22 ins F	ACCGGTTAATAGTACCTGTG	P-EbfG::YFP, PCR analysis on *E. coli* cells
NSI- pRP22 ins R	ATGCCCGCGACATCTTCC	
NSI and pRP22 F	AGACGAGGCAAGCATTGAGC	P-EbfG::YFP, PCR analysis on cyano cells
NSI- pRP22 ins R	ATGCCCGCGACATCTTCC	
**Cloning of fragments encoding EbfG proteins into C-DAG system[3]**		
**Primer name**	**Primer sequence (upper forward, lower reverse)**	**Purpose**
pExport:ebfg1	TATAGCGGCCGCAAAATTCTGGGGG	PCR amplification
	TATATCTAGATTAGTGATGATGGTGATGGTGGGCAGATACAGTTC	
pExport:ebfg2	TATAGCGGCCGCAAGTGGTTATTCCATT	PCR amplification
	TATATCTAGATTAGTGATGATGGTGATGGTGAGCTGAAACAGTC	
pExport:ebfg3	TATAGCGGCCGCAAAATTCTGGGGG	PCR amplification
	TATATCTAGATTAGTGATGATGGTGATGGTGGGCTGTAACTGC	
pExport:ebfg4	TATAGCGGCCGCAACTAATTGCAATCC	PCR amplification
	TATATCTAGATTAGTGATGATGGTGATGGTGTTGCGATTATATTTCTT	
pExport	CCTGACGCTTTTTATCG	Sequencing
	GCTGAAAATCTTCTCTC	

Supplementary table 1: Summary of cloning information

[1] EbfG4::FLAG tagged vectors were generated using the GeneArt® Seamless Cloning and Assembly Kit (Life Technologies) and Top10 cells (Life Technologies) on three PCR-generated fragments per plasmid and a SwaI-digested CYANO-VECTOR cloning plasmid, pCV0049 or pAM4937, that encodes for kanamycin (Km) resistance, Neutral Site II (NS2) integration, and

a *ccdB*-suicide gene that is removed upon SwaI digestion [1]. PCR fragments were amplified from the complementation plasmid pAM4997 (pRP10, [2]) using Q5® High-Fidelity DNA polymerase (New England Biolabs) and primers designed to add overlaps for cloning into the SwaI cut site or to add the flag tags to the C-terminus of the encoded EbfG4 protein. Clones were screened by PCR and the entire complementation region of the newly generated vectors were verified by Sanger sequencing.

[2] The genomic region bearing the promoter of the *ebfG*-opreon and YFP were PCR amplified and then served as primers for each other to PCR amplify a fusion product that was inserted in a neutral site 1 in the chromosome [1].

[3] PCR products were amplified from pRP10 [2] and cloned into the vector pExport [3, 4]. Fragments were inserted at the NotI (5') and XbaI (3') sites between the CsgA secretion signal and stop codon of pExport. All cloning products were validated by PCR analyses and sequencing.

Unprocessed images

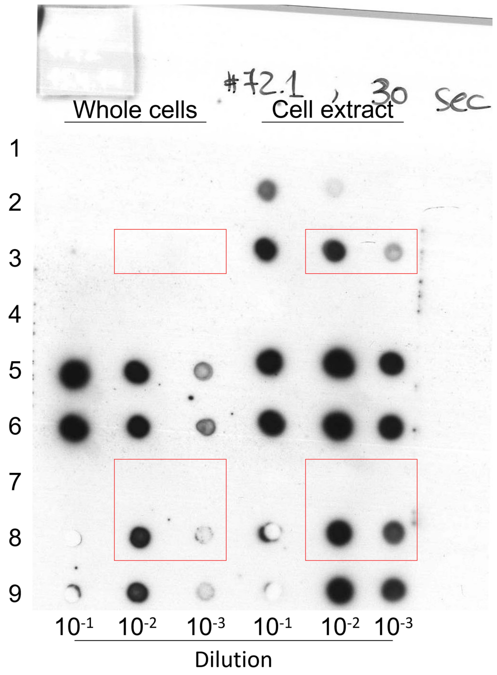

Unprocessed image for figure 3: Analysis includes the following strains: 1. PilB::Tn5 (negative control), 2&3 Hfq-mutant complemented with Hfq::FLAG and PilB::Tn5 complemented with PilB::FLAG, respectively (positive controls for intracellular proteins). 4. *ebfG*-operon-deleted strain into which EbfG1::FLAG was introduced; 5&6 the latter strain that also harbors *pilB* inactivation (two different transformants); 7. *ebfG4*-inactivated strain complemented with FLAG-tagged EbfG4 (EbfG4Ω/EbfG4::FLAG); 8&9 the latter strain that also harbors *pilB* inactivation (PilB::Tn5/EbfG4Ω/EbfG4::FLAG) (two different transformants). Regions presented in Fig.3 are indicated by red rectangles. Explanatory notes for data selection: For Fig. 3 we selected one positive control (3) and strains harboring EbfG4::FLAG without (7) and with (8) *pilB*-inactivation. Dilution 10^{-1} is not presented in Fig. 3 because for PilB::Tn5/EbfG4Ω/EbfG4::FLAG strains (8&9) data show an artifact of Enhanced Chemiluminescence detection known as “ghost band”. These white areas result from rapid substrate depletion due to excess antigen and conjugated antibody. Initial experiments included strains with FLAG-tagged EbfG1, however, data for these strains are not included in Fig. 3 because fluorescence microscopy analyses were focused on an EbfG4::FLAG strain.

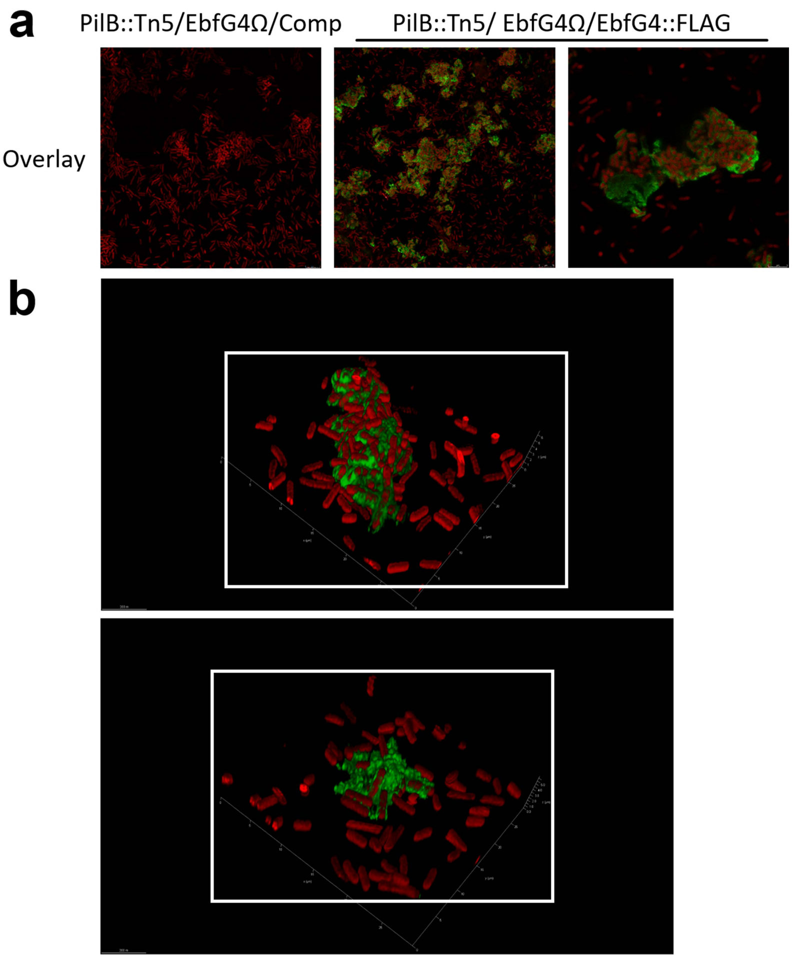

Unprocessed image for figure 4: a. The complete recruited fields are included in Fig. 4. Contrast adjustment was applied to the whole image. b. Rectangles indicate cropped area.

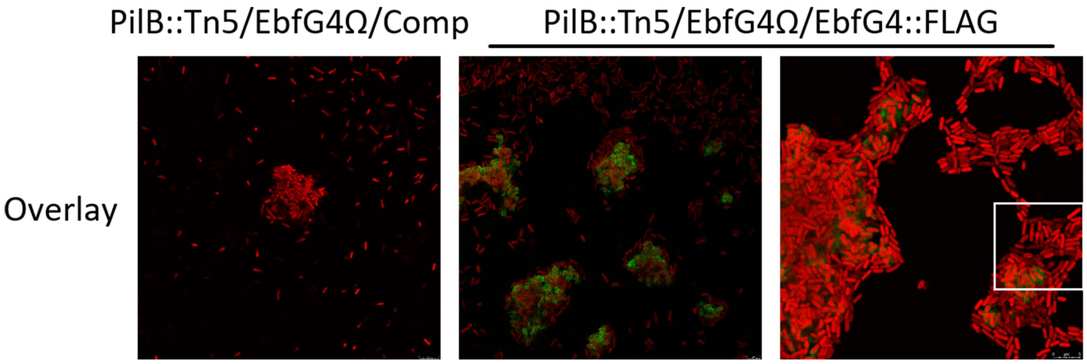

Unprocessed image for figure 5: Left and middle panels: Complete recruited fields are included in Fig. 5. Contrast adjustment was applied to the whole image. Right Panel: Rectangle indicates cropped area.

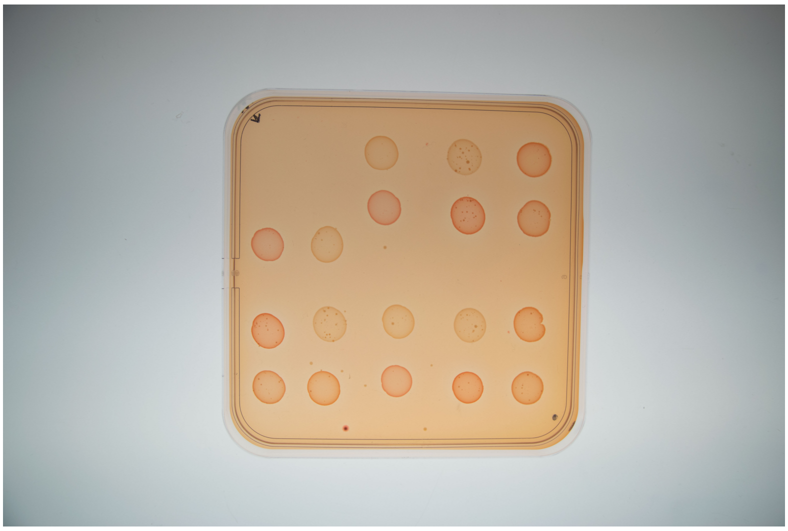

Unprocessed image for figure 6b: The colonies were cropped to include one of the three technical replicates and the brightness adjusted to better differentiate the colors.

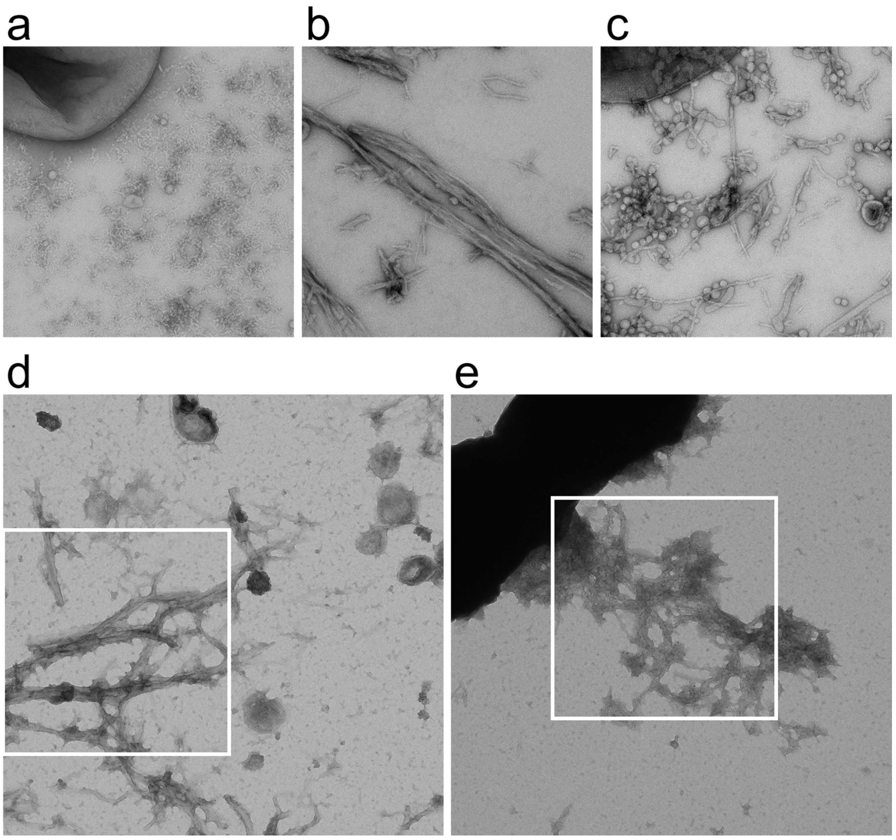

Unprocessed images for figure 7:
Panels d and e were cropped to focus on gold particles. Rectangle indicates cropped area.

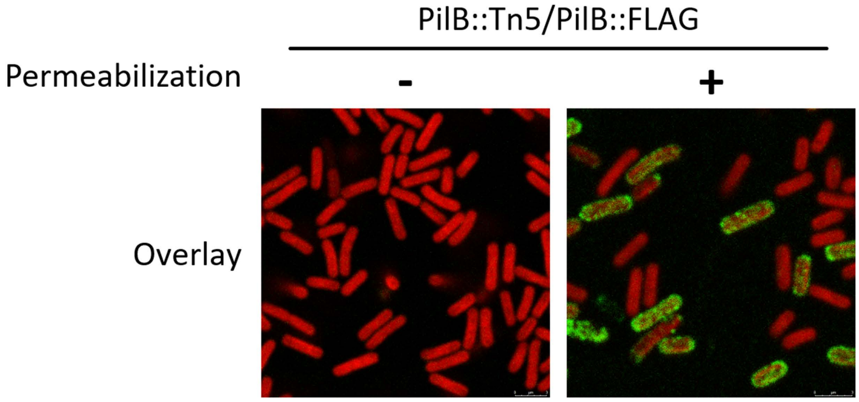

Unprocessed image for supplementary figure 4: The complete recruited fields are included in supplementary Fig. 4. Adjustments were not applied to these images.

Supplementary References

1. Taton, A., et al., *Broad-host-range vector system for synthetic biology and biotechnology in cyanobacteria.* Nucleic Acids Res, 2014. **42**(17): p. e136.
2. Parnasa, R., et al., *Small secreted proteins enable biofilm development in the cyanobacterium Synechococcus elongatus.* Sci Rep, 2016. **6**: p. 32209.
3. Sivanathan, V. and A. Hochschild, *A bacterial export system for generating extracellular amyloid aggregates.* Nat Protoc, 2013. **8**(7): p. 1381-90.
4. Sivanathan, V. and A. Hochschild, *Generating extracellular amyloid aggregates using E. coli cells.* Genes & Development, 2012. **26**(23): p. 2659-2667.
