# Supplementary material for: Cell specialization in cyanobacterial biofilm development revealed by expression of a cell-surface and extracellular matrix protein
Source: NPJ Biofilms Microbiomes. 2023 Mar 2;9:10. doi: 10.1038/s41522-023-00376-6 (PMC9981879; doi:10.1038/s41522-023-00376-6)
Supplement: Supplementary file 2 — Reporting Summary [file 41522_2023_376_MOESM2_ESM.pdf]

nature portfolio

Corresponding author(s): Rakefet Schwarz

Last updated by author(s): Dec 6, 2022

Reporting Summary

Nature Portfolio wishes to improve the reproducibility of the work that we publish. This form provides structure for consistency and transparency in reporting. For further information on Nature Portfolio policies, see our Editorial Policies and the Editorial Policy Checklist.

Statistics

For all statistical analyses, confirm that the following items are present in the figure legend, table legend, main text, or Methods section.

n/a	Confirmed	
☐	☒	The exact sample size (n) for each experimental group/condition, given as a discrete number and unit of measurement
☐	☒	A statement on whether measurements were taken from distinct samples or whether the same sample was measured repeatedly
☐	☒	The statistical test(s) used AND whether they are one- or two-sided<br>*Only common tests should be described solely by name; describe more complex techniques in the Methods section.*
☐	☒	A description of all covariates tested
☐	☒	A description of any assumptions or corrections, such as tests of normality and adjustment for multiple comparisons
☐	☒	A full description of the statistical parameters including central tendency (e.g. means) or other basic estimates (e.g. regression coefficient) AND variation (e.g. standard deviation) or associated estimates of uncertainty (e.g. confidence intervals)
☐	☒	For null hypothesis testing, the test statistic (e.g. F, t, r) with confidence intervals, effect sizes, degrees of freedom and P value noted<br>*Give P values as exact values whenever suitable.*
☒	☐	For Bayesian analysis, information on the choice of priors and Markov chain Monte Carlo settings
☒	☐	For hierarchical and complex designs, identification of the appropriate level for tests and full reporting of outcomes
☒	☐	Estimates of effect sizes (e.g. Cohen's d, Pearson's r), indicating how they were calculated

Our web collection on statistics for biologists contains articles on many of the points above.

Software and code

Policy information about availability of computer code

Data collection	No software was used
Data analysis	Flowcore package version 2.6.0; lmerTest package version 23.21-20; Emmeans R package version 1.7.1-1; LASX Viewer version 3.5.5; Huygens software version 18.04; TANGO with default parameters version 2.2; APPNN version 1.0; AmyloGram version 1.1; Custom code pipeline: https://github.com/danielzmbp/amypred; Statistical sequence analysis, WALTZ with default parameters webserver; ArchCandy version 1.0; Pasta version 2.0; APPNN version 1.0; PATH version 1.1; Metamyl webserver; Cordax webserver; ChimeraX version 1.3

For manuscripts utilizing custom algorithms or software that are central to the research but not yet described in published literature, software must be made available to editors and reviewers. We strongly encourage code deposition in a community repository (e.g. GitHub). See the Nature Portfolio guidelines for submitting code & software for further information.

Data

Policy information about availability of data

All manuscripts must include a data availability statement. This statement should provide the following information, where applicable:

- Accession codes, unique identifiers, or web links for publicly available datasets
- A description of any restrictions on data availability
- For clinical datasets or third party data, please ensure that the statement adheres to our policy

The data underlying this article are available within the article and the accompanying Supplementary Information. Additional data are available from the corresponding author upon request.

Human research participants

Policy information about studies involving human research participants and Sex and Gender in Research.

Reporting on sex and gender	Not relevant
Population characteristics	Not relevant
Recruitment	Not relevant
Ethics oversight	Not relevant

Note that full information on the approval of the study protocol must also be provided in the manuscript.

Field-specific reporting

Please select the one below that is the best fit for your research. If you are not sure, read the appropriate sections before making your selection.

☒ Life sciences ☐ Behavioural & social sciences ☐ Ecological, evolutionary & environmental sciences

For a reference copy of the document with all sections, see nature.com/documents/nr-reporting-summary-flat.pdf

Life sciences study design

All studies must disclose on these points even when the disclosure is negative.

Sample size	For flow cytometry experiments ~10000 cells per sample were recorded and all data were analyzed. For other analyses, as commonly accepted, three biological independent experiments were performed (see details in "Replication").
Data exclusions	No data were excluded expect for rare cases in which contamination with heterotrophic bacteria was detected during the course of the experiment.
Replication	To confirm reproducibility, at least three biological independent experiments were performed (with three technical repeats). Cyanobacterial cultures were grown in controlled environment as described in Methods.
Randomization	Mutants for characterization and cognate control cultures were placed randomly in the growth room.
Blinding	Not relevant for biofilm assays as phenotype is obvious from culture appearance. For analysis of flow cytometry data, statistician received only coded labels. For fluorescence microscopy analysis, samples were coded.

Reporting for specific materials, systems and methods

We require information from authors about some types of materials, experimental systems and methods used in many studies. Here, indicate whether each material, system or method listed is relevant to your study. If you are not sure if a list item applies to your research, read the appropriate section before selecting a response.

Materials & experimental systems

n/a	Involved in the study
☐	☒ Antibodies
☒	☐ Eukaryotic cell lines
☒	☐ Palaeontology and archaeology
☒	☐ Animals and other organisms
☒	☐ Clinical data
☒	☐ Dual use research of concern

Methods

n/a	Involved in the study
☒	☐ ChIP-seq
☐	☒ Flow cytometry
☒	☐ MRI-based neuroimaging

Antibodies

Antibodies used: anti-FLAG, ab1162, Abcam; goat anti rabbit IgG, 170-6515, Bio-Rad; goat anti-rabbit Alexa Fluor 488, Abcam; anti-polyhistidine, mouse peroxidase-coupled IgG2, A7058, Sigma Aldrich

Validation: Cell/extracts not possessing the epitope served as negative controls. Samples for which previous studies confirmed the presence of the epitome served as positive controls.

Flow Cytometry

Plots

Confirm that:

☒ The axis labels state the marker and fluorochrome used (e.g. CD4-FITC).

☒ The axis scales are clearly visible. Include numbers along axes only for bottom left plot of group (a 'group' is an analysis of identical markers).

☒ All plots are contour plots with outliers or pseudocolor plots.

☒ A numerical value for number of cells or percentage (with statistics) is provided.

Methodology

Sample preparation: 50 ml culture at exponential phase was centrifuged (6000g, room temperature), resuspended with 4 ml fresh BG11 to obtain a concentrated culture for inoculation into fresh medium or CM at an OD750 of 0.5. Aliquots of 0.5 ml were taken from each culture tube following 6 days of growth and then, in case of biofilm-forming strains, planktonic cells were removed. 1.5 ml BG11 were used to resuspend the biofilmed cells by rigorous pipettation and 0.13 ml were transferred to a 1.5 ml Eppendorf tube for homogenization with a pellet pestle (Sigma-Aldrich, Z359971-1EA). The homogenized samples were filtered through a mesh (pore size 52 µm), supplemented with formaldehyde to a final concentration of 1%, diluted with phosphate-buffered saline (PBS) to OD750 of ~0.0001. For each sample, ~10000 cells were analysed.

Instrument: BD FACSAria

Software: All statistical analyses were conducted in the statistical program R, version 3.3.2. FCS files obtained from FlowJo were analyzed with flowcore package (version 2.6.0). Mean, median, and robust coefficient of variation (CV) of the intensity distribution for each sample were calculated. Robust CV was calculated as defined in the FlowJo documentation https://docs.flowjo.com/flowjo/workspaces-and-samples/ws-statistics/ws-statdefinitions/. Intensity values were log-transformed. Significant difference between biofilm and planktonic cells of a particular culture was tested using Paired t-tests on several intensity distribution parameters (mean, median and robust CV). Initial analysis did not reveal significant differences between biofilm and planktonic cells within a particular culture, therefore, these data were combined for further analysis. Effect of growth medium or genetic background on intensity distribution parameters (mean, median and robust CV) was tested with 2-way repeated measures ANOVA. Specifically, mixed linear effect models were fitted with medium or genetic background as fixed effects and biological replicates as random effect, (using lmerTest package version 23.21-20), and the ANOVA was performed on the resulting models. Post hoc pairwise comparisons were performed by testing linear contrasts (using emmeans R package version 1.7.1-1), and FDR correction was applied to control for multiple testing. Normality of residuals and homogeneity of variances assumptions were checked graphically.

Cell population abundance: Not relevant

Gating strategy: Gating is based on cyanobacterial autofluorescence (see Fig. S7).

☒ Tick this box to confirm that a figure exemplifying the gating strategy is provided in the Supplementary Information.
